# Evaluation of Antioxidant and Hepatoprotective Activities of *Moringa oleifera* Lam. Leaves in Carbon Tetrachloride-Intoxicated Rats

**DOI:** 10.3390/antiox3030569

**Published:** 2014-09-02

**Authors:** Dharmendra Singh, Priya Vrat Arya, Ved Prakash Aggarwal, Radhey Shyam Gupta

**Affiliations:** 1 Centre for Advanced Studies, Department of Zoology, University of Rajasthan, Jaipur 302 055, India; E-Mail: d.singh2009@gmail.com; 2 Department of Zoology, Dyal Singh College, University of Delhi, Lodhi Road, New Delhi 110 003, India; E-Mails: zoology.dsc@gmail.com (P.V.A.); vpa541@gmail.com (V.P.A.)

**Keywords:** antioxidants, free radicals, hepatoprotection, *Moringa oleifera*

## Abstract

The antioxidant and hepatoprotective activities of the extract of *Moringa oleifera* leaves were investigated against CCl_4_-induced hepatotoxicity in rats. Hepatotoxic rats were treated with ethanol extract of *Moringa oleifera* for a period of 60 days at the following three dose levels; 100, 200 and 400 mg/kg body weight/day, orally. The activities were studied by assaying the serum marker enzymes like SGOT, SGPT, GGT, LDH, ALP, ACP, as well as total bilirubin, total protein and albumin in serum concomitantly with the activities of LPO, SOD, CAT, GSH, GR and GPx in liver. The activities of all parameters registered a significant (*p* ≤ 0.001) alteration in CCl_4_ treated rats, which were significantly recovered towards an almost normal level in rats co-administered with *M. oleifera* extract in a dose-dependent manner. All the biochemical investigations were confirmed by the histopathological observations and compared with the standard drug. silymarin. Results suggest that the antioxidant and hepatoprotective activities of *M. oleifera* leaves are possibly related to the free radical scavenging activity which might be due to the presence of total phenolics and flavonoids in the extract and/or the purified compounds β-sitosterol, quercetin and kaempferol, which were isolated from the ethanol extract of *M. oleifera* leaves.

## 1. Introduction

*Moringa oleifera* Lam. is a drumstick tree of the Moringaceae family, known as sahinjan in Hindi. It is a native of India and now grown extensively in many southeastern Asian countries particularly in Thailand, Philippines and Pakistan *etc.* [1]. Traditionally, *Moringa oleifera* is considered to be one of the most useful trees in the world, as almost every part of the tree has some nutritional, medicinal and other valuable properties [2]. The leaves, especially young shoots, are eaten as salads, in vegetable curries, and as pickles. Additionally, the shade dried leaves of *Moringa oleifera* are widely utilized in developing countries as a good source of protein, calcium, vitamin A, C and E, β-carotene, amino acids, various polyphenolics and some natural anti-oxidizing agents [3]. Therefore, it is used as an alternative source of nutritional supplements and growth promoters in various countries [4].

Apart from nutritional benefits, *Moringa oleifera* has been extensively used as an antioxidant, and for the following applications; wound healing, anti-tumor, anti-fertility, hypotensive, antipyretic, antihepatotoxic, antiepileptic, anti-inflammatory, antiulcer, diuretic, hypocholesterolaemic, antifungal, antibacterial and anti-cardiovascular agent, * etc.* [5,6]. The extract of *Moringa oleifera* leaves is also capable of reducing hyperglycemia, dyslipidemia [7], and diabetes mellitus [8]. Hence, the present study was carried out in an attempt to evaluate the antioxidant and hepatoprotective activities of *M. oleifera* leaves extract in carbon tetrachloride CCl_4_-intoxicated rats which might be due to the presence of total phenolics and flavonoids in the extract and/or isolated active constituents β-sitosterol, quercetin and kaempferol.

## 2. Material and Methods

### 2.1. Plant Material

Fresh leaves of *Moringa oleifera* Lam. were collected from the University Campus, University of Rajasthan, Jaipur and authenticated by the Department of Botany, University of Rajasthan, Jaipur (Herbarium Sheet No. RUBL-20287).

### 2.2. Extraction and Isolation

The leaves were shade-dried and pulverized. The powder was treated with petroleum ether for defatting as well as to remove chlorophyll. The powder was packed into a soxhlet apparatus and subjected to hot continuous percolation using alcohol (95% v/v) as solvent for 48 h at 58–60 °C. The extract was concentrated under vacuum, dried in a vacuum desiccator, and yielded 7.4% w/w of a dark greenish-brown solid mass. The solid mass was then powdered, and washed with chloroform to remove the remaining chlorophyll content present in the extract. Half of the extract was suspended in an appropriate volume of olive oil to prepare the desired concentration for oral administration to rats during experimentation.

The rest of the extract (30 g) was subjected to traditional column chromatography by fractionation with different solvents. For this purpose, a column (1.4 m × 5 cm) filled with 900 g Si-gel (60–120 mesh) was used. The purity of fractions was checked by qualitative thin layer chromatography using different solvent systems. After ascertaining the purity of compounds, it was subjected to detailed spectral analysis (IR, ^1^H NMR, ^13^C NMR and MS) to establish the structure. As a result, the compounds (A, B & C) were isolated, purified and characterized.

#### 2.2.1. Isolation and Characterization of Compound A

Compound A was obtained when the column was eluted with chloroform and benzene in the ratio 1:1. After removal of solvent, A was obtained as yellowish crystalline solid. Further purification by re-column chromatography yielded β-sitosterol, colorless needles, 1.8 g/30 g (6% w/w), R_f_ 0.56(CHCl_3_)_3_ m.p. 136–137 °C and responded positively to the Liebermann-Burchard test for sterols. It was identified by a mixed melting point and Co-TLC with an authentic sample. IR (ν_max_) cm^−1^ (KBr): 3450, 2940, 2880, 1600, 1040; ^1^HNMR (δ ppm, CDCl_3_): 0.65 (3H, s), 0.85 (3H, s), 0.90 (3H, s), 0.95 (3H, s), 0.98 (3H, s), 1.25 (3H, s), 1.45 to 1.85 (for 26H, 10CH_2_ and 6CH), 1.85 (H, d), 2.05 (1H, d), 2.25 (1H, d), 3.50 (1H, m), 5.30 (1H, d); ^13^CNMR (δ ppm, CDCl_3_): 31.3(C-1), 31.7(C-2), 71.8(C-3), 42.3(C-4), 141.0(C-5), 121.7(C-6), 31.9(C-7), 45.9(C-8), 50.1(C-9), 36.1(C-10), 21.1(C-11), 28.2(C-12), 42.3(C-13), 56.8(C-14), 24.3(C-15), 39.8(C-16), 56.1(C-17), 11.8(C-18), 19.3(C-19), 36.1(C-20), 19.0(C-21), 36.1(C-22), 24.3(C-23), 39.8(C-24), 28.2(C-25), 23.1(C-26), 23.1(C-27), 31.9(C-28), 29.2(C-29); MS *m*/*z*: 414(M^+^), 399, 383, 369, 256, 215, 174, 159, 145, 134, 120, 107, *etc.*

**Figure 1:**
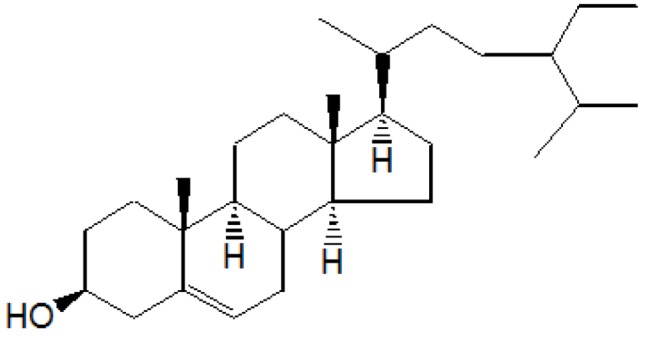
Compound A.

On the basis of ^1^HNMR, ^13^CNMR and mass spectral studies of compound A, it was characterized as β-sitosterol (Figure 1) with molecular formula C_29_H_50_O and comparable with the literature of β-Sitosterol [9,10].

#### 2.2.2. Isolation and Characterization of Compound B

A light brown solid was obtained after removal of the solvent of acetone and methanol (1:1) as light yellow needles, 2.85 g/30 g (9.5% w/w), m.p. 301–302 °C. The compound was insoluble in pet ether, sparingly soluble in benzene, ethyl acetate and acetone, soluble in methanol and NaOH solution. It gave a blue-green color with alcoholic FeCl_3_, a reddish brown colour in Shinoda’s test and a yellow color showing light green fluorescence with conc. H_2_SO_4_ indicating its flavonoid nature. It gave a dark bright yellow spot on the TLC plate when viewed in UV light. IR (ν_max_) cm^−1^ (KBr): 3450–3100 (broad), 3040, 3010, 1660, 1590, 1520, 1230, 1200, 1180, 910, 830, 820 and 790; ^1^HNMR (δ ppm, CDCl_3_ + DMSO-d_6_): 12.31 (OH, s), 10.25 (OH, s), 8.87 (OH, s), 8.68 (OH, s), 8.31 (OH, s), 7.81–7.62 (2H, m), 7.00 (1H, d), 6.50 (1H, d) and 6.32 (1H, d); MS *m*/*z*: 302(M^+^), 301,235, 284, 152, 137, 135, 132, 105, 95, 89, 77, *etc*.

**Figure 2:**
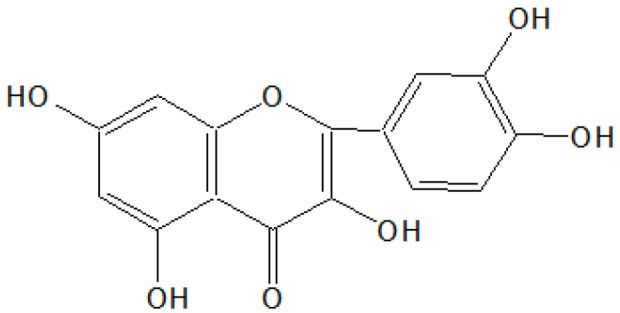
Compound B.

On the basis of ^1^HNMR and mass spectral studies of compound B, it was characterized as quercetin (Figure 2) with molecular formula C_15_H_10_O_7_ and comparable with the literature of quercetin [9,11].

#### 2.2.3. Isolation and Characterization of Compound C

The methanol fraction afforded a greenish-yellow solid after removal of solvent, which was recrystallized with aqueous ethanol as light yellow needles, 4.95 g/30 g (16.5% w/w), m.p. 274–276 °C. It dissolved in alkali, to give a green colour with alcoholic FeCl_3_ and a fluorescent blue colour with conc. H_2_SO_4_. It gave the positive Shinoda’s test characteristic for flavonoids (to a pinch of substance in 1 mL alcohol, magnesium and a few drops of conc. HCl were added) where it gave a dark red color. In UV light on TLC plates it displayed a yellow color spot with an R_f_ value 0.95 in the following system; ethyl acetate: formic acid: glacial acetic acid: water (51:11:11:27). IR (ν_max_) cm^−1^ (KBr): 3380, 3050, 3020, 1680, 1590, 1490, 1410, 1220, 1150, 910, 830, and 780; ^1^HNMR (δ ppm, CDCl_3_ + DMSO-d_6_): 12.25 (1H, s), 10.18 (1H, s), 9.56 (1H, s), 8.12 (2H, d), 7.62 (1H, s), 6.93 (2H, d), 6.43 (1H, d) and 6.25 (1H, d); MS *m*/*z*: 286(M^+^), 285, 269, 268, 152, 121, 105, 95, 89, 77, *etc.*

**Figure 3:**
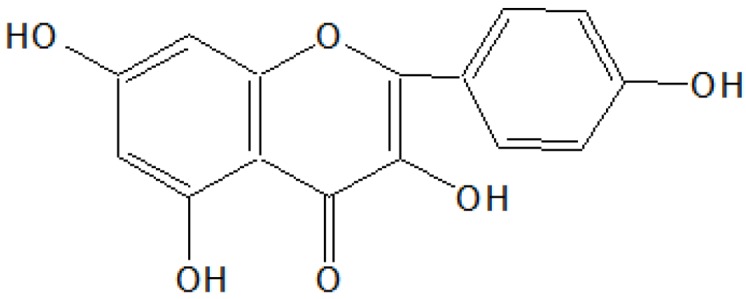
Compound C.

On the basis of ^1^HNMR and mass spectral studies of compound C, it was characterized as kaempferol (Figure 3) with molecular formula C_15_H_10_O_6_ and comparable with the literature of kaempferol [11,12].

### 2.3. Silymarin

Silymarin was purchased from MP Biomedicals, France and it was dissolved in olive oil for oral administration to rats during experimentation at the dose level-25 mg/kg body weight/day [13].

### 2.4. Chemicals

All chemicals were analytical grade and chemicals required for all biochemical assays were obtained from Sigma Chemicals Co., St. Louis, MO, USA.

### 2.5. Animal Model

Colony bred healthy, adult male albino rats (wistar strain) (*Rattus norvegicus*) weighing 175–200 g were used in the present study. The rats were housed in polypropylene cages under controlled conditions of temperature (23–26 °C), humidity (60%–70%) and light (12 h light/dark cycle). They were provided with a nutritionally adequate standard laboratory diet (Lipton, India Ltd., Bangalore, India) and tap water *ad libitum*.

### 2.6. Ethical Aspects

The study was approved by the ethical committee (Protocol No. 1678/Go/a/12/CPCSEA/73) of the University Department of Zoology, Jaipur, India. Indian National Science Academy, New Delhi, (INSA, 2000) guidelines were followed for maintenance and use of the experimental animals.

### 2.7. Chronic Toxicity

The extract of plant material was administered to all the test groups in graded doses ranging up to 3 g/kg body weight and the rats were observed for signs of toxicity and mortality for 60 days afterward. The extract was found to be practically non-toxic when given orally to rats and its LD_50_ value was found to be higher than 3 g/kg body weight [data not shown]. The minimum dose levels viz. 100, 200 and 400 mg/kg body weight were used for oral administration to rats during experimentation [14].

### 2.8. Treatment Design

After acclimatization of 15 days, the animals were divided into the following groups containing 06 animals in each group:

Group I: Vehicle treated rats were kept on normal diet and served as control for 60 days.
Group II: Rats were intoxicated with carbon tetrachloride (CCl_4_) at the dose level of 0.3 mL/kg body weight/twice a week, intra-peritoneally for 60 days.
Group III: Rats orally received *M. oleifera* extract at 100 mg/kg body weight/day, and CCl_4_ as Group II for 60 days, simultaneously.
Group IV: Rats orally received *M. oleifera* extract at 200 mg/kg body weight/day, and CCl_4_ as Group II for 60 days, simultaneously.
Group V: Rats orally received *M. oleifera* extract at 400 mg/kg body weight/day, and CCl_4_ as Group II for 60 days, simultaneously.
Group VI: Rats orally received silymarin (as a reference standard drug) at 25 mg/kg body weight/day and CCl_4_ as Group II for 60 days, simultaneously.

### 2.9. Autopsy Schedule

After the last dose-delivery, rats of each group were kept on starvation for 24 h and after that anaesthetized under mild ether anesthesia. Blood samples were collected by cardiac puncture. The blood samples of each animal were taken and allowed to clot at 37 °C and the serum was separated by centrifugation then stored at 4 °C until assayed.

After the collection of blood, the liver was immediately excised, washed with cold normal saline, blotted, and weighed on an electrical balance. Half of the liver was fixed in Bouin’s fixative for histological studies and the remaining half was immediately frozen (at −20°/−70 °C) for biochemical analysis.

### 2.10. Analysis and Processing of the Samples

The biochemical analysis of enzymes in serum samples *viz.* serum glutamic oxaloacetic transaminase (SGOT), serum glutamic pyruvic transaminase (SGPT), gamma glutamyl transpeptidase (GGT), lactate dehydrogenase (LDH), alkaline phosphatase (ALP), acid phosphatase ACP) and total bilirubin, total protein and albumin were performed using kit methods. SGOT (Batch No. 61105), SGPT (Batch No. 60865) and GGT (Batch No. 34004) kits were purchased from Accurex Biomedical Pvt. Ltd.; Mumbai, India. LDH (Lot. No. 6854), ALP (LOT. No.7093), ACP (LOT. No.6666), total bilirubin (LOT. No. 6801), total protein (LOT. No. 6808) and albumin (LOT. No. 6988) kits were purchased from Span Diagnostics Ltd., Surat, India, respectively.

In tissue samples, a part of liver tissue was minced and homogenized in ice-cold 1.15% w/v KCl in a Potter Elvehjem Teflon glass homogenizer for 1 min to make a 10% w/v liver homogenate. Lipid peroxidation (LPO) [15] was measured in the liver homogenate. The quantitative estimation of hepatic antioxidant enzymes such as superoxide dismutase (SOD) [16], catalase (CAT) [17], reduced glutathione (GSH) [18], glutathione reductase (GR) [19], and glutathione peroxidase (GPx) [20] were performed in liver homogenate also, respectively.

### 2.11. Histopathology

Liver was fixed in Bouin’s fixative for 24 h and after that dehydrated in ethanol series (50%–100%), cleared in xylene, and embedded in paraffin using the standard microtechnique. Sections of the liver (5 μm) were stained with alum haematoxylin and eosin (H-E) for histopathological changes.

### 2.12. Statistical Analysis

Statistical analysis was performed using one-way analysis of variance (ANOVA) followed by student “*t*” test. The values are mean ± S.E. for six rats in each group. *p* values ≤0.05 were considered as significant.

## 3. Results

**Figure 4:**
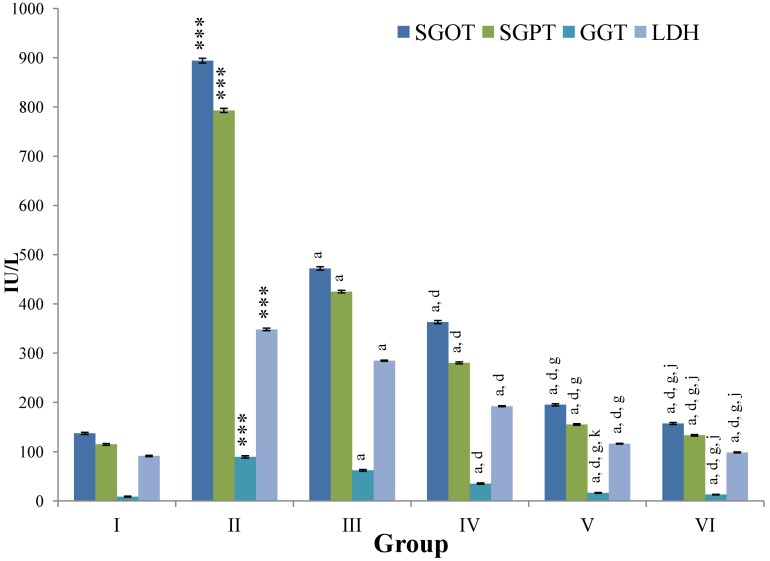
Changes in the activity of serum glutamic oxaloacetic transaminase (SGOT), serum glutamic pyruvic transaminase (SGPT), gamma glutamyl transpeptidase (GGT), lactate dehydrogenase (LDH) in serum after various treatments of *M. oleifera* leaves extract and silymarin in rats. Data points with different symbol and letter notations are significantly different at *** *p* ≤ 0.001: Group II compared to Group I, ^a^ *p* ≤ 0.001: Group III–VI compared to Group II, ^d^ *p* ≤ 0.001: Group IV–VI compared to Group III, ^g^ *p* ≤ 0.001: Group V & VI compared to Group IV, ^j^ *p* ≤ 0.001; ^k^ *p* ≤ 0.01: Group VI compared to Group V.

**Figure 5:**
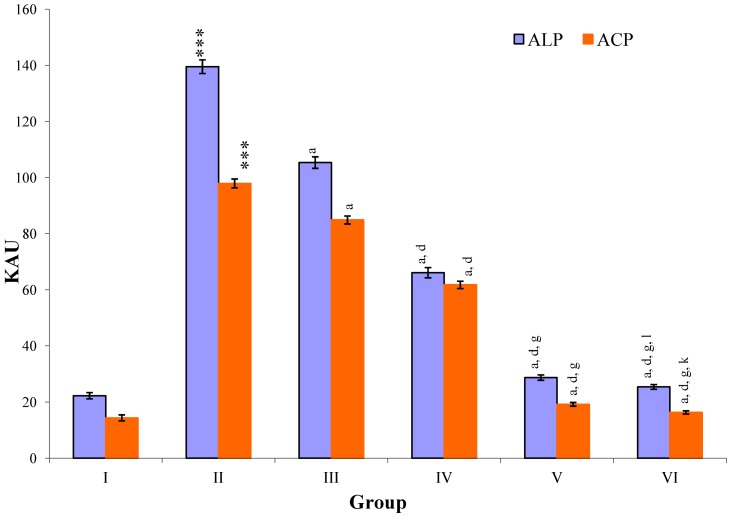
Changes in the activity of alkaline phosphatase (ALP), and acid phosphatase (ACP) in serum after various treatments of *M. oleifera* leaves extract and silymarin in rats. Data points with different symbol and letter notations are significantly different at ^***^ *p* ≤ 0.001 Group II compared to Group I, ^a^ *p* ≤ 0.001: Group III–VI compared to Group II, ^d^ *p* ≤ 0.001: Group IV–VI compared to Group III, ^g^ *p* ≤ 0.001: Group V & VI compared to Group IV, ^k^ *p* ≤ 0.001; ^1^ *p* ≤ 0.05: Group VI compared to Group V.

**Figure 6:**
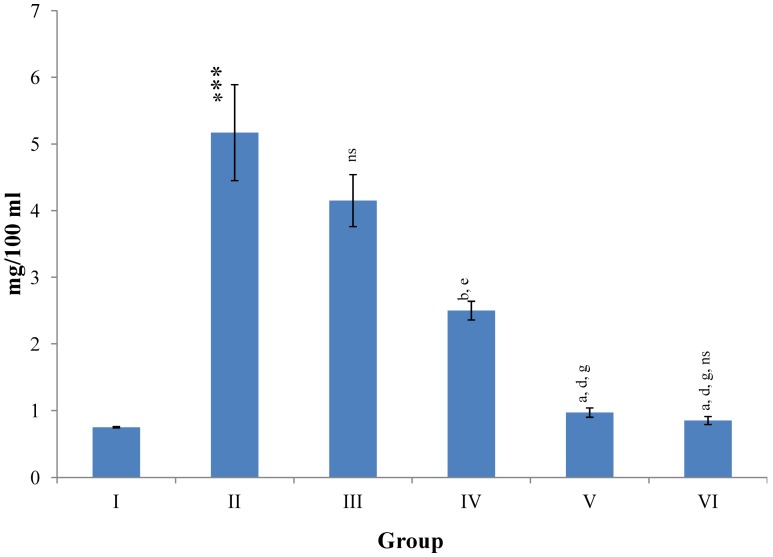
Changes in the total bilirubin concentration in serum after various treatments of *M. oleifera* leaves extract and silymarin in rats. Data points with different symbol and letter notations are significantly different at ^***^ *p* ≤ 0.001 Group II compared to Group I, ^a^ *p* ≤ 0.001; ^b^ *p* ≤ 0.001; ^ns^ non significant: Group III–VI compared to Group II, ^d^ *p* ≤ 0.001: Group IV–VI compared to Group III, ^g^ *p* ≤ 0.001: Group V & VI compared to Group IV, ^ns^ non significant: Group VI compared to Group V.

**Figure 7:**
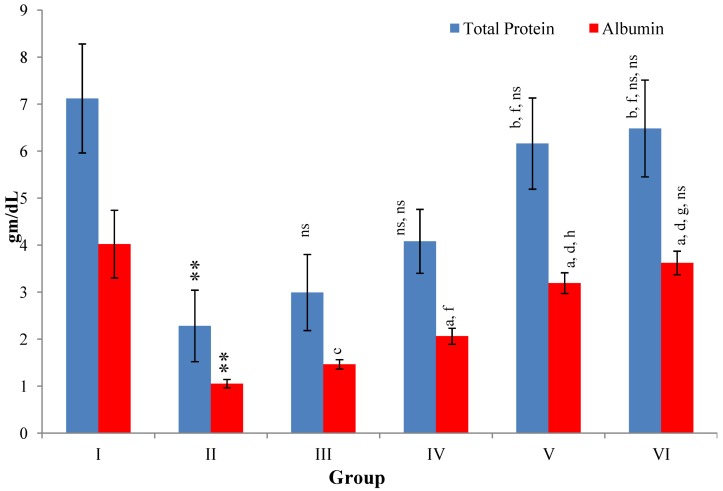
Changes in the total protein and albumin in serum after various treatments of *M. oleifera* leaves extract and silymarin in rats. Data points with different symbol and letter notations are significantly different at ^**^ *p* ≤ 0.01 Group II compared to Group I, ^a^ *p* ≤ 0.001; ^b^ *p* ≤ 0.01; ^c^ *p* ≤ 0.05; ^ns^ non significant: Group III–VI compared to Group II, ^d^ *p* ≤ 0.001; ^f^ *p* ≤ 0.05; ^ns^ non significant: Group IV–VI compared to Group III, ^g^ *p* ≤ 0.001; ^h^ *p* ≤ 0.01; ^ns^ non significant: Group V & VI compared to Group IV, ^ns^ non significant: Group VI compared to Group V.

CCl_4_-intoxication to rats for the period of 60 days resulted in a significant (*p* ≤ 0.001) rise in the levels of SGOT, SGPT, GGT, LDH, ALP, ACP and total bilirubin along with notable reduction (*p* ≤ 0.01) in the levels of total protein and albumin in serum when compared with normal controls (Figure 4, Figure 5, Figure 6 and Figure 7).

The effect of *M. oleifera* leaves extract on serum—SGOT, SGPT, GGT, LDH, ALP, ACP and total bilirubin in CCl_4_-induced rats—was found to be reduced significantly (*p* ≤ 0.001) along with remarkable elevation in the total protein and albumin at all three dose levels (Group III–V) as compared to CCl_4_-treated Group II. The dose level of 400 mg/kg of *M. oleifera* was most effective but the attenuation of altered serum parameters was not as high as in silymarin treated rats of Group VI (Figure 4, Figure 5, Figure 6 and Figure 7).

**Figure 8:**
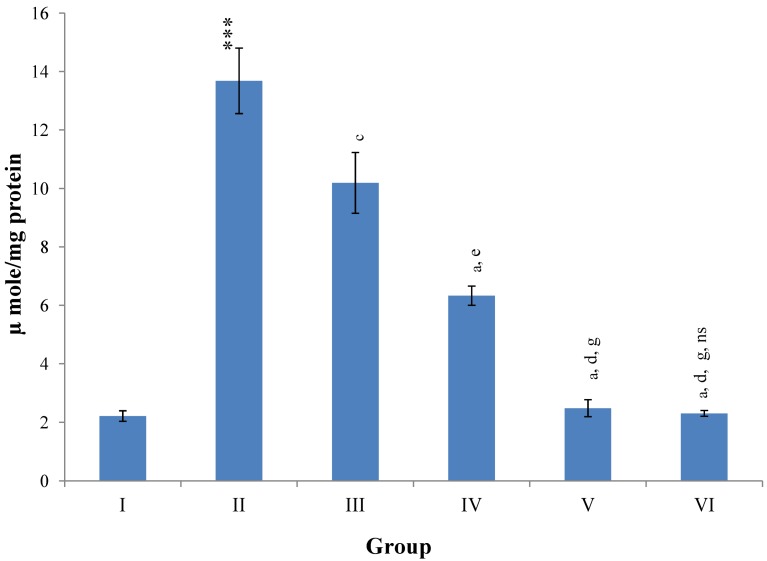
Changes in the lipid peroxidation in liver after various treatments of *M. oleifera* leaves extract and silymarin in rats. Data points with different symbol and letter notations are significantly different at *** *p* ≤ 0.001: Group II compared to Group I, ^a^ *p* ≤ 0.001; ^c^ *p* ≤ 0.05: Group III–VI compared to Group II, ^d^ *p* ≤ 0.001; ^e^ *p* ≤ 0.01: Group IV–VI compared to Group III, ^g^ *p* ≤ 0.001: Group V & VI compared to Group IV, ^ns^ non significant: Group VI compared to Group V.

Figure 8 illustrates a significant (*p* ≤ 0.001) increase in the level of hepatic LPO in CCl_4_ intoxicated rats as compared to normal controls. Treatment with *M. oleifera* leaves extract significantly (*p* ≤ 0.05; *p* ≤ 0.001) prevented this rise in LPO level at all three dose levels (Group III–V). The silymarin treated group VI showed non-significant decline in the level of hepatic LPO as compared to higher dose level (400 mg/kg) of *M. oleifera* extract treated rats (Group V).

**Figure 9:**
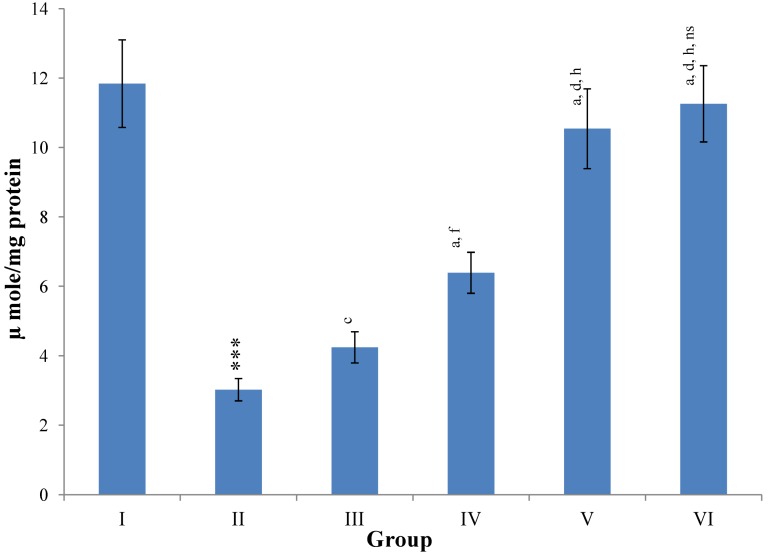
Changes in the superoxide dismutage (SOD) in liver after various treatments of *M. oleifera* leaves extract and silymarin in rats. Data points with different symbol and letter notations are significantly different at *** *p* ≤ 0.001: Group II compared to Group I, ^a^ *p* ≤ 0.001; ^c^ *p* ≤ 0.05: Group III–VI compared to Group II, ^d^ *p* ≤ 0.001; ^f^ *p* ≤ 0.05: Group IV–VI compared to Group III, ^h^ *p* ≤ 0.01: Group V & VI compared to Group IV, ^ns^ non significant: Group VI compared to Group V.

**Figure 10:**
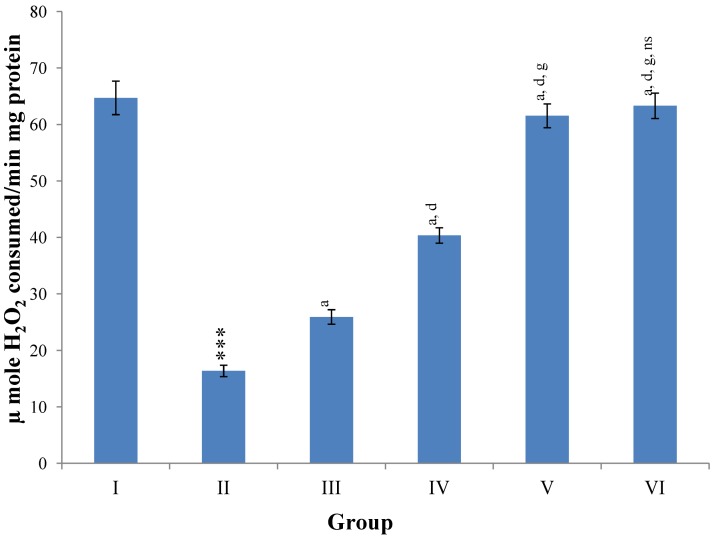
Changes in the catalase in liver after various treatments of *M. oleifera* leaves extract and silymarin in rats. Data points with different symbol and letter notations are significantly different at *** *p* ≤ 0.001: Group II compared to Group I, ^a^ *p* ≤ 0.001: Group III–VI compared to Group II, ^d^ *p* ≤ 0.001: Group IV–VI compared to Group III, ^g^ *p* ≤ 0.001: Group V & VI compared to Group IV, ^ns^ non significant: Group VI compared to Group V.

**Figure 11:**
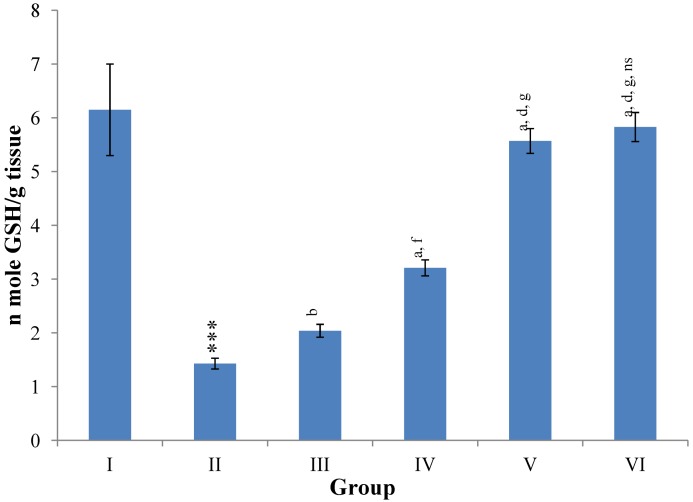
Changes in the glutathione (reduced) in liver after various treatments of *M. oleifera* leaves extract and silymarin in rats. Data points with different symbol and letter notations are significantly different at *** *p* ≤ 0.001: Group II compared to Group I, ^a^ *p* ≤ 0.001; ^b^ *p* ≤ 0.01: Group III–VI compared to Group II, ^d^ *p* ≤ 0.001; ^f^ *p* ≤ 0.05: Group IV–VI compared to Group III, ^g^ *p* ≤ 0.001: Group V & VI compared to Group IV, ^ns^ non significant: Group VI compared to Group V.

**Figure 12:**
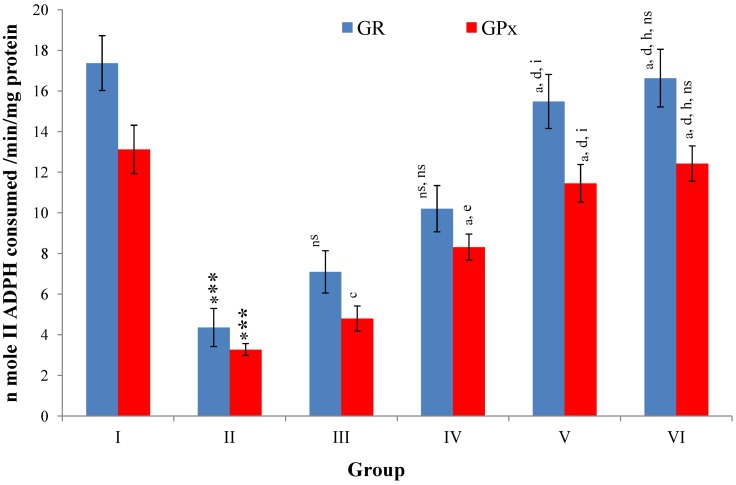
Changes in the glutathione reductase (GR) and glutathione peroxidase (GPx) in liver after various treatments of *M. oleifera* leaves extract and silymarin in rats. Data points with different symbol and letter notations are significantly different at *** *p* ≤ 0.001: Group II compared to Group I, ^a^ *p* ≤ 0.001; ^c^ *p* ≤ 0.05; ^ns^ non significant: Group III–VI compared to Group II, ^d^ *p* ≤ 0.001; ^e^ *p* ≤ 0.01; ^ns^ non significant: Group IV–VI compared to Group III, ^h^ *p* ≤ 0.01; ^i^ *p* ≤ 0.05: Group V & VI compared to Group IV, ^ns^ non significant: Group VI compared to Group V.

Hepatic SOD, CAT, GSH, GR and GPx contents were elevated significantly (*p* ≤ 0.001) in extract treated animals at 400 mg/kg dose level (Group V) but also showed non-significant to significant (*p* ≤ 0.05; *p* ≤ 0.01; *p* ≤ 0.001 respectively) elevation in the levels of all the above antioxidant parameters at 100 and 200 mg/kg dose levels (Group III and VI) whereas CCl_4_-intoxicated Group II was shown to have a highly significant (*p* ≤ 0.001) decrease in the all above antioxidant contents as compared to normal controls. Therefore, the dose levels of 400 mg/kg of *M. oleifera* extract also exhibited again as a most effective dose but showed a non-significant elevation compared to silymarin treated rats of Group VI (Figure 9, Figure 10, Figure 11 and Figure 12).

**Figure 13:**
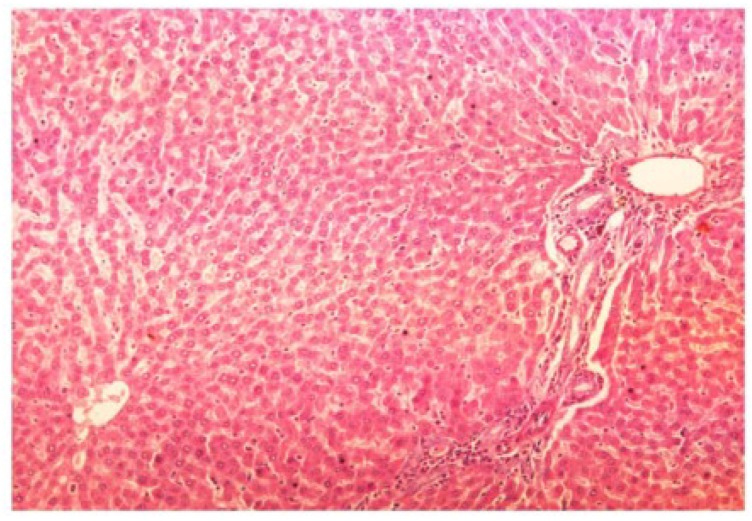
Photomicrograph of control rat liver section (60 days) showing well brought central vein, hepatic cells with preserved cytoplasm and prominent nucleus at H & E × 100.

**Figure 14:**
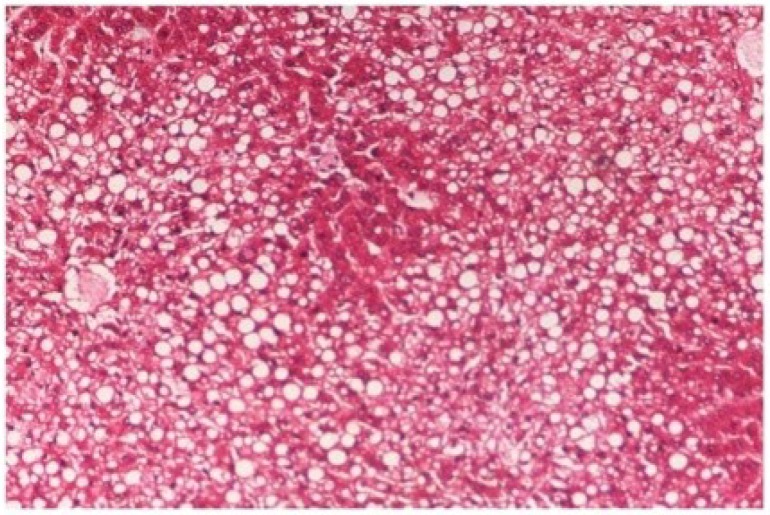
Photomicrograph of rat liver section with CCl_4_ treatment (60 days) showing marked steatosis of the hepatocytes with ballooning degeneration and distended portal veins and necrosis at H & E × 100.

**Figure 15:**
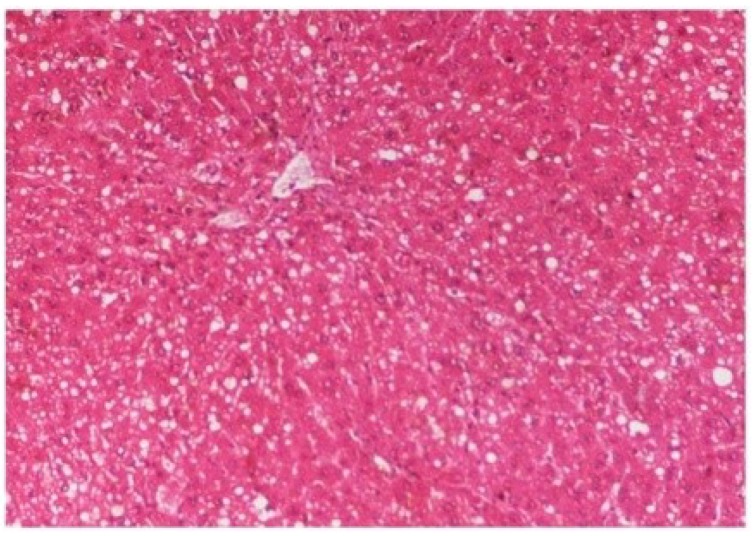
Photomicrograph of rat liver section of CCl_4_ + *M. oleifera* extract at 100 mg/kg (60 days) showing slight reduction in fatty degenerative changes with congestion of sinusoids, necrosis and infiltration of polymorphonucleocytes at H & E × 100.

**Figure 16:**
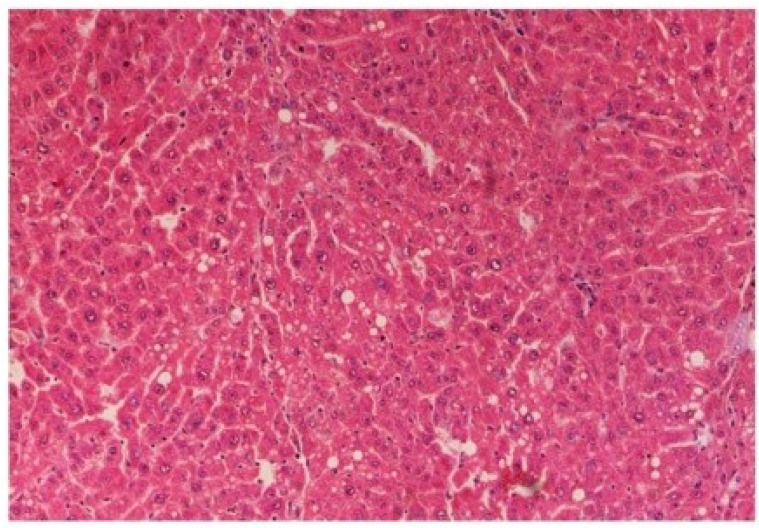
Photomicrograph of rat liver section of CCl_4_ + *M. oleifera* extract at 200 mg/kg (60 days) showing considerable reduction in necrosis and fatty changes with pyknotic nuclei and cytoplasmic clearing at H & E × 100.

**Figure 17:**
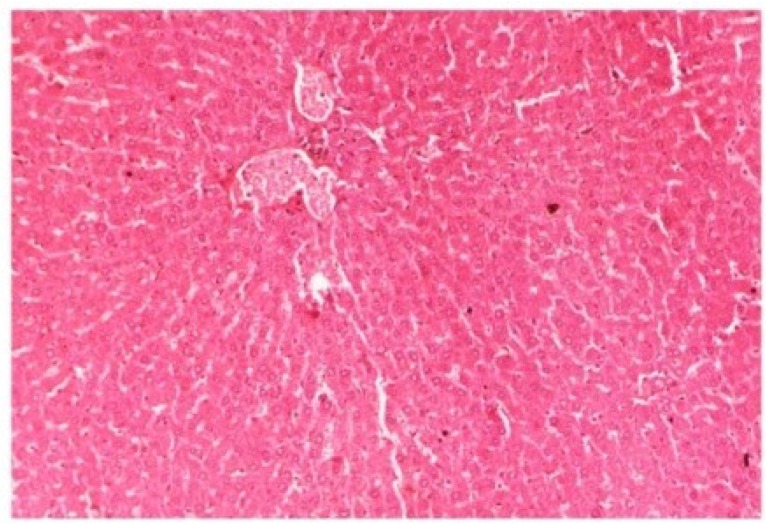
Photomicrograph of rat liver section of CCl_4_ + *M. oleifera* extract at 400 mg/kg (60 days) showing moderately brought central vein, hepatic cells with preserved cytoplasm and prominent nucleus at H & E × 100.

**Figure 18:**
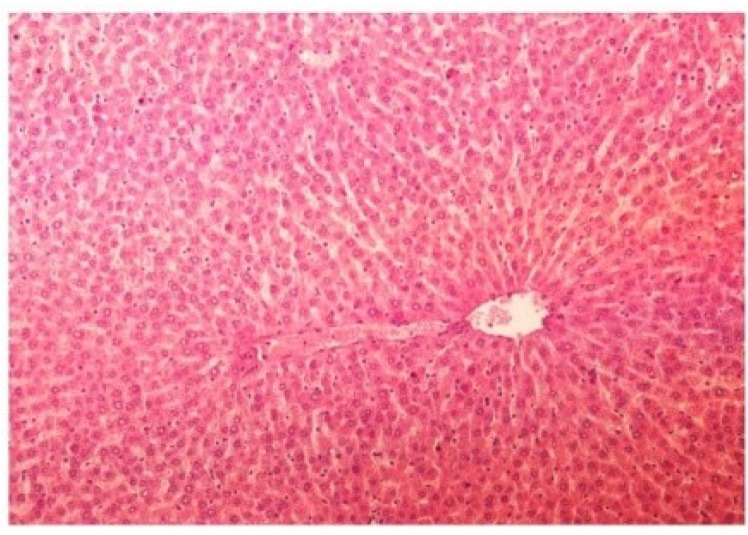
Photomicrograph of rat liver section of CCl_4_ + silymarin at 25 mg/kg (60 days) depicting histological pattern almost similar to liver of normal rats with preserved cytoplasm and prominent nucleus at H & E × 100.

The histopathology of CCl_4_-intoxicated rats when compared with normal hepatic architecture (Figure 13) showed massive fatty changes, necrosis, ballooning degeneration and the loss of cellular boundaries (Figure 14). The liver sections of *M. oleifera* extract at 100 mg/kg dose level plus CCl_4_-treated rats (Group III) showed mild prevention of CCl_4_-induced degenerative changes with the few pyknotic nuclei and fatty vacuolization in cytoplasm (Figure 15). The liver sections of extract treated rats at the dose level of 200 mg/kg along with CCl_4_ (Group VI) indicated partial amelioration of degenerative effects in hepatocytes but still showed cloudy swelling and mild fatty changes (Figure 16). Histomorphological picture of liver sections of *M. oleifera* extract at the dose level of 400 mg/kg along with CCl_4_-induction to rats (Group V) showed more or less normal lobular pattern with the devoid of degenerative changes and preserved cytoplasm with prominent nucleus without intracellular lipid accumulation (Figure 17) almost comparable to the normal control and silymarin treated Group VI (Figure 18).

## 4. Discussion

It is generally accepted in the scientific world that the hepatotoxicity induced by CCl_4_ is due to the formation of the active metabolite, trichloromethyl free radical (CCl_3_^•^). This then readily interacts with molecular oxygen to form the trichloromethyl peroxy radical (CCl_3_OO^•^). Both radicals are capable of binding to proteins and other macromolecules with simultaneous attack on poly-unsaturated fatty acids to produce lipid peroxidation leading to hepatotoxicity [21].

Lipid peroxidation of hepatic cell membrane is one of the principle causes of hepatic injury induced by CCl_4_ or other hepatotoxicants. This is because lipid peroxidation is viewed as a complicated biochemical reaction involving free radicals, metal ions, oxygen and a host of many different factors in the biological system. Also in recent years, lipids and their derivatives have been recognized as important molecules in signal transduction [22].

The efficacy of any liver curative drug depends on its capacity of either reducing the harmful effect or restoring the normal hepatic physiology that has been made anxious by CCl_4_ and/or other hepatotoxicants [23]. In our present study, the measurement of LPO in the liver tissue is a convenient method to monitor oxidative cell damage. Inhibition of elevated LPO has been observed in *M. oleifera* extract and silymarin treated groups due to its antioxidant and free radical scavenging activities through re-establishment of biomembranes of hepatic parenchymal cells [24].

In agreement with results obtained in previous investigations [25,26], our present study elicited a significant increase in the activities of SGOT, SGPT, GGT, LDH, ALP, and ACP with the exposure to CCl_4_ which is indicated by cellular leakage and loss of functional integrity of the hepatic cell membrane [27,28]. Oral treatment with *M. oleifera* extract and silymarin attenuated these increased enzyme activities produced by CCl_4_ and a subsequent recovery towards normalization of these enzymes strongly suggests the possibility of *M. oleifera* extract being able to affect the hepatocytes so as to cause accelerated regeneration of parenchymal cells and lysosomes, thus protecting against lysosomal integrity and cell membrane fragility, and therefore decreasing the leakage of marker enzymes into the circulation [25]. Stabilization of serum total bilirubin level by the administration of *M. oleifera* extract is further a clear signal of the improvement of the functional status of the hepatic cells [29].

The significant reduction in the total protein and albumin levels in serum with exposure to CCl_4_ causes considerable liver damage through induction of peroxidation of lipids and finally inhibits the protein synthesis due to trichloromethyl free radical covalent bindings [30]. The treatment with *M. oleifera* extract and silymarin stabilized the serum total protein and albumin levels. The stabilization of proteins might be considered as an indication of enhanced protein synthesis in the hepatic cells due to inhibition of peroxidation of lipids and scavenge of the free radicals [31].

The antioxidant defense mechanisms include enzymatic and non-enzymatic antioxidants playing a significant role in the sustaining of physiological levels of O_2_ and H_2_O_2_ and eradicating the peroxides generated from inadvertent exposure to toxic drugs. Any natural medications with antioxidant profiles may help to maintain health when continuously taken as components of dietary food, spices or remedies. Among the antioxidant enzymes, SOD and catalase are the first line of defense against CCl_4_ induced hepatic oxidative damage. SOD is the primary step of the defense mechanism in the antioxidant system against oxidative stress by catalyzing the dismutation of superoxide radicals (O^2−^) into molecular oxygen (O_2_) and hydrogen peroxide (H_2_O_2_). H_2_O_2_ is neutralized by the action of catalase [32]. A significant depletion in the activities of hepatic SOD and catalase during CCl_4_ intoxication to rats might be due to the enhanced superoxide radical formation leading to oxidative stress in the tissue. Administration of *M. oleifera* extract to CCl_4_ treated rats enhanced the SOD and catalase profiles, dose-dependently, by acting as a strong free radical quencher and protecting the hepatic cells. Therefore SOD and catalase are essential for the endogenous antioxidative defense system to scavenge reactive oxygen species and maintain the cellular redox balance [33,34].

GSH is one of the most abundant tripeptide, non-enzymatic biological antioxidants present in the hepatocytes, which is a key component of the overall antioxidant defense system that protects the membrane protein thiols of hepatocytes from deleterious effects of reactive oxygen metabolites such as hydrogen peroxide and superoxide radicals [35]. The decline of GSH level in the CCl_4_ treated group might be due to its utilization by the excessively generated quantity of free radicals in the hepatocytes leading to hepatic injury. However, the subsequent recovery in rats treated with *M. oleifera* extract might be due to *de-novo* GSH synthesis or GSH regeneration (GSSG to GSH), dose-dependently.

After the CCl_4_ induced oxidative liver damage, GPx along with catalase metabolize H_2_O_2_ to water and other non-toxic substances. This antioxidant system also consists of GSH and a range of functionally interrelated enzymes, of which GR and GPx are responsible for the regeneration of GSH or from GSSG to GSH, where both enzymes work together with GSH in the decomposition of hydrogen peroxide and also other biological hydroperoxides [36]. The dose-dependent reversal of GR and GPx activity to near normal level with the *M. oleifera* extract treatment showed the antioxidant activity of plant extract by scavenging the endogenous metabolic peroxides generated after CCl_4_ induced damage in the liver tissue.

Histopathological observations suggested that the reactive oxygen species and lipid peroxidation may play an important role in pathogenesis of hepatocytes as hepatic fibrosis and necrosis with the loss of normal liver architecture. Because of CCl_4_ toxicity, a toxic reactive metabolite trichloromethyl free radical was produced which binds covalently to macromolecules of the lipid membranes of adipose tissue and causes peroxidative degradation. As a result, fats from the adipose tissue are translocated and accumulated in the hepatocytes [24,36,37]. The degenerative changes were shown to be minimal or absent with the plant extract treatment, dose-dependently. This might be due to lower fat accumulation and re-establishment of the antioxidant defense system in the liver tissue through the antioxidant and hepatoprotective nature of *M. oleifera* leaves.

## 5. Conclusions

The antioxidant and hepatoprotective potential of *M. oleifera* leaves extract may be attributed to the presence of total phenolics and flavonoids in the extract and/or isolated active constituents—β-sitosterol, quercetin and kaempferol—which have hydroxyl group(s). The hydroxyl group(s), because of its resonance property, easily donates e^−^ to free radicals and effectively neutralizes them. Also, the presence of a hydroxyl group(s) increases its antioxidant potential through intermolecular hydrogen bonding involving the -SH group of non-protein thiols and enzymes resulting in the restoration of the antioxidant system against oxidative damage in mammalian liver tissue.
